# The impact of different rehabilitation strategies after major events in the elderly: the case of stroke and hip fracture in the Tuscany region

**DOI:** 10.1186/1472-6963-7-95

**Published:** 2007-06-27

**Authors:** Fabrizio Carinci, Lorenzo Roti, Paolo Francesconi, Rosa Gini, Fabrizio Tediosi, Tania Di Iorio, Simone Bartolacci, Eva Buiatti

**Affiliations:** 1 Agenzia Regionale di Sanità della Toscana, Firenze, Italy; 2 Serectrix Health Systems Research, Pescara, Italy; 3 Swiss Centre for International Health, Basel, Switzerland

## Abstract

**Background:**

On a regional level, our aims were to describe rehabilitation patterns for elderly patients with stroke and hip fracture and to investigate mortality risk during the 6-month post acute period.

**Methods:**

Data sources included administrative data relative to patients aged 65+ resident in Tuscany admitted in hospital for stroke or hip fracture between 2001 and 2003, traced up to 3 years before and 6 months following index admission. The study design involves computerized linkage of administrative data, and an exploratory analysis of the association between rehabilitation patterns and 6-month mortality, adjusting for clinical, demographic, and acute-related care characteristics using multivariate Cox regression.

**Results:**

Rehabilitation patterns vary greatly across Tuscany with considerable cost implications. Six month mortality risk for stroke patients is significantly lower among residents of Local Health Authorities where patients are more frequently rehabilitated, specifically in extra-hospital settings.

**Conclusion:**

Our study, targeting two crucial conditions for elderly patients, found a high variability of rehabilitation patterns across a region, albeit coherent between the two pathologies, associated with remarkable differences in average expenditure. Differences in hazard rates for 6-month mortality after stroke at population level were also found. These results need to be confirmed and further investigated through a more robust information framework.

## Background

National health systems increasingly need to monitor the impact of their policies in order to optimise care. European countries need to identity new solutions for the emerging needs caused by multiple chronic conditions affecting the elderly, frequently as a direct consequence of acute events. Stroke and hip fracture represent major risk factors for the onset of progressive and catastrophic disability [1] and high-impact triggers of a range of complications known to be significantly associated with increased mortality [2-4].

Both conditions typically require a mix of medical/rehabilitative services such as inpatient, outpatient and home-based care, whose integration needs to be carefully monitored [5]. Despite all efforts, there is a large variation in terms of utilization, costs, and outcomes of rehabilitation in the elderly following an acute event [2,6-11]. Due to the expanding availability of standardised databases, it is now possible to use computerized data-linkage to conduct a system-wide evaluation of rehabilitation.

Aims of the study are to describe patterns of utilization of rehabilitation services in Tuscany for subjects aged 65 and older, using stroke and hip fracture as index conditions, and to investigate their association with post-acute 6-month mortality.

## Methods

### The Regional Health Care System

The study was conducted in the region of Tuscany, Central Italy, with a population of approximately 3.5 million, 23% aged 65+ and 11% over 74. The regional health care system is organized in 12 Local Health Authorities (LHAs) and 4 independent Hospital Trusts (HT). LHAs organize all health services – hospital care included – while HTs provide highly specialized care. According to the latest regional directive in the field of rehabilitation [12], after an acute episode patients can undergo residential and semi-residential rehabilitation care, either in hospital rehabilitation wards, or extra-hospital rehabilitation facilities, outpatient or home rehabilitation care, with services provided by multidisciplinary teams at extra-hospital rehabilitation facilities or by individual physiotherapists. Regional guidelines also identify three phases of rehabilitative care following acute events: rehabilitation provided during the stay in the acute hospital ward (for stays longer than 10 days only); intensive rehabilitation, provided soon after discharge from the acute hospital ward for 30–40 days in the different settings; and extensive rehabilitation, provided, if needed, after intensive rehabilitation by community care services or through admission in a nursing home.

### Data Sources

The available regional administrative databases (hospital discharges, outpatient specialist services, extra-hospital rehabilitation services) and the regional mortality register were linked through a personal unique identifier (tax file number). An *ad hoc *algorithm was used to check the results of the linkage procedure. Residents in one of the 12 LHAs were excluded from the final analysis due to inaccurate recording.

### Study Population

The study population refers to residents in Tuscany aged at least 65, discharged from acute hospital wards between 1/7/2001 and 30/6/2003 with a primary diagnosis of stroke (ICD-9-CM 430, 431, 434, 436) [13-16], never admitted for stroke during the previous three years, or with a primary diagnosis of hip fracture (ICD-9-CM 820–821) [17], not admitted with the same diagnosis during the previous 28 days (index admissions).

### Study Variables

Selected demographic, clinical, acute care-related and ecological rehabilitation variables were identified as potential correlates of mortality. Demographic characteristics included age, gender and marital status. Clinical characteristics included major comorbidities, according to diagnoses available from hospital admissions up to three years prior to the index event [18-21], type of stroke (ischemic stroke, subarachnoid haemorrage and intracerebral haemorrhage) and type of hip fracture (intracapsular, extracapsular, other or unspecified). Acute care-related factors included length of stay (LOS) at index admission, admission in stroke unit (for stroke), surgery procedure within 2 days of admission (for hip fracture). Surgery procedures were identified using standard criteria (ICD-9-CM codes 7905, 7915, 7925, 7935, 7945, 7955, 8151, 8152). Stroke units were identified according to previously defined criteria [22]. We also defined five rehabilitation settings according to the first service provided after discharge from the acute hospital ward: Inpatient Hospital (residential rehabilitation care in hospital rehabilitation wards); Inpatient Facility (residential rehabilitation care in extra-hospital rehabilitation facilities); Outpatient Rehabilitation (semi-residential and outpatient rehabilitation care); Home Rehabilitation; and Hospital Stay with Rehabilitation Procedures (rehabilitation procedures provided in non-rehabilitation acute hospital wards during an hospital admission occurring after discharge from the index admission).

### Statistical Analysis

Univariate statistics and graphical outputs were used to describe rehabilitation services provided after the index event. Percentages are expressed in relation to either the total population experiencing the index event (including in-hospital deaths), or, when appropriate, to the subjects discharged alive. We investigated the association between rehabilitation patterns and 6-month mortality through the definition of an ecological variable classifying LHAs by the average use of rehabilitation services for their residents. A specific plot was used to position LHAs (displayed as a dot with a unique alpha ID) on a bivariate plan showing the proportion of patients rehabilitated after discharge (X axis) *vs *the proportion of subjects undergoing inpatient rehabilitation among those rehabilitated (Y axis). A superimposed grid was used to define four quadrants based on the median values of the proportion of rehabilitated patients overall and in hospital: (I) both below the median, (II) overall below the median and in hospital above the median, (III) overall above the median and in hospital below the median, and (IV) both above the median.

Proportional hazards regression [23] was the basis of multivariate survival analysis. Multiple observations per patient were used to take into account time varying covariates. Times at risk were computed starting from the 3^rd ^quartile of LOS in the acute wards. This way we excluded early deaths, both in-hospital and post-discharge not relevant for post-acute rehabilitation, while defining a common start-up for the follow-up of all patients. Multivariate Cox regression analysis was used to evaluate the independent association between rehabilitation and increased mortality, adjusting for all other individual characteristics. Final models were produced forcing in all variables considered clinically meaningful as fixed effects, including individual-level covariates (demographic, clinical and acute care related factors) and an ecological rehabilitation variable [24]. All analyses were performed using STATA [25], version 8.2.

### Ethics and consent

Data processing and statistical analysis have been conducted at the Regional Offices of Agenzia Regionale di Sanità della Toscana. As a technical and scientific structure of the Regional Government, the Agenzia has the right to use administrative databases and publish summary reports (Regional Law n.28 10^th^ July 2006, Art.3.1.1.b) and is granted direct access to identifiable individual health data (Art.19.1.2).

## Results

**Figure 1:**
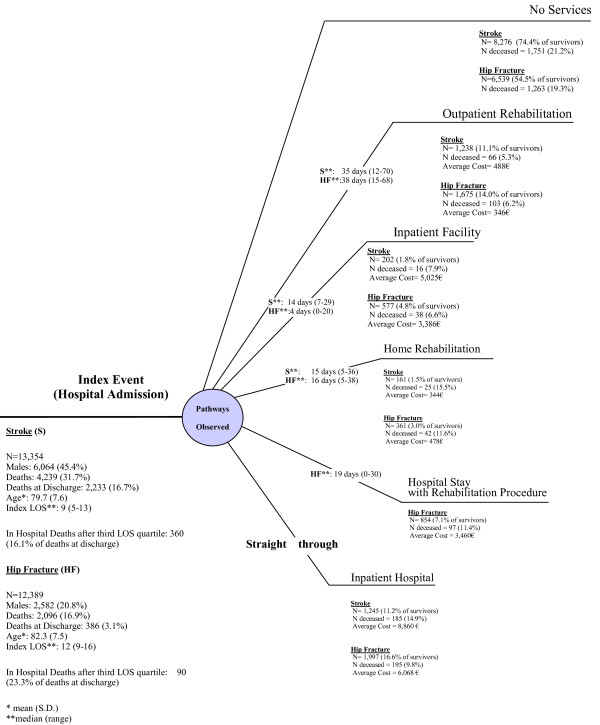
Rehabilitation Patterns after Stroke/Hip Fracture, 12 LHAs, Tuscany Jul 01–Jun 03.

Population under study (N = 12 LHAs), rehabilitation settings and 6-month mortality rates are described in Figure 1. Out of the 13,354 subjects with stroke identified in the reference time interval, 16.7% died during the index admission (83.9 % of whom before the 3^rd ^quartile LOS) and further 15.0% within 180 days after discharge. For hip fracture, 12,389 subjects were extracted, with 3.1% deaths recorded in hospital (76.7 % of whom before the 3rd quartile LOS) and further 13.8% within 180 days after discharge. Stroke patients were slightly younger than subjects experiencing hip fractures, with a higher prevalence of males. Average LOS was longer among subjects with hip fractures, albeit less variable. In terms of acute care, 549 patients with stroke (4.1%) were admitted to stroke units while 2,801 patients with hip fracture (22.6%) underwent surgery within 2 days from admission. Overall, a lower proportion of stroke patients discharged alive underwent some form of rehabilitation compared to same with hip fracture (25.6 *vs *45.5%), but the rehabilitated patients (with the exception of rehabilitation procedures in ordinary acute care, identified only for hip fracture patients) were similarly distributed in the rehabilitation settings across the two events. Also the median delay between discharge and rehabilitation were similar across the two events, except for inpatient facility care provided to hip fracture patients with a median delay of 4 days vs 2 weeks for stroke. There was no delay for hospital rehabilitation, 15–16 days for home-based care, 35–38 days for outpatient care.

In both cases, risk of death during the six-month follow-up was higher for patients who did not undergo any rehabilitation (approx 20%), intermediate for subjects rehabilitated in hospital or at home (between 10–15%), lower for those rehabilitated in outpatient or inpatient settings (below 5%).

In stroke, the average economic value of rehabilitation services per rehabilitated subject was 8,860€ for inpatient rehabilitation, 5,025 € for inpatient facility, 489€ for outpatient and 344€ for home-based care. Average economic values for hip fracture were 25% lower for each service, although similarly more expensive for inpatient care than other solutions.

**Figure 2:**
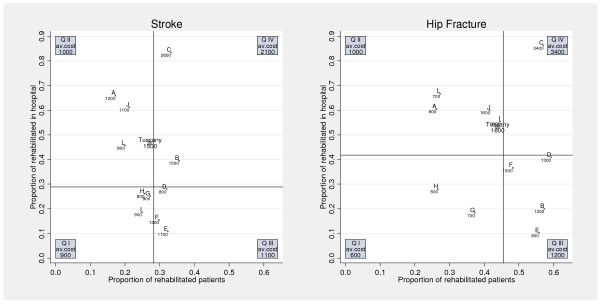
Observed proportion of rehabilitated patients (out of those discharged alive) and of rehabilitated in hospital (out of those rehabilitated) among residents in 11 LHAs in Tuscany discharged alive for stroke/hip fracture between 1/7/01 and 30/6/03. Dots are LHAs with alphabetic ID and average cost per discharged patient. Label box: quadrant ID and average cost.

Figure 2 shows the variability across the region based on 11 LHAs with accurate data. There is a large variation in the proportions of patients undergoing rehabilitation, and of those rehabilitated in hospital among those rehabilitated, with considerable cost implications. Variation across LHAs is two-fold for the average proportion of rehabilitated patients and eight-fold for the proportion of subjects rehabilitated that has received rehabilitation in hospital. The majority of dots in the plot lie in the same quadrant across the two diseases: only two LHAs (B, I) appear under different quadrants, with just one (I) crossing non-adjacent ones, i.e. classifying differently according to both variables. Such variability is also reflected by economic values, showing a three-fold to seven-fold variation in the mean estimated cost of rehabilitation services per patient discharged alive for stroke and hip fracture respectively. Remarkably, LHAs in quadrant IV present by far the highest costs, almost double (for stroke) or triple (for hip fracture) those of LHAs in quadrant III.

Table 1 presents the general demographic, clinical, acute-care and ecological rehabilitation characteristics of N = 10,622 subjects with stroke and N = 11,720 subjects with hip fracture, resident in the 11 LHAs entering the analysis, alive at the 3^rd ^quartile of the LOS after index admission.

**Table 1:** General Characteristics of patients admitted for Stroke (S) and Hip Fracture (HF)

***Dimension***	***Stroke***		***Hip Fracture***	
**Variable**	**Entering analysis**	**Dead within 6 months**	**Entering analysis**	**Dead within 6 months**

*Categories*				

**N**	10622	1383 (13.0)	11720	1407 (12.0)
***Demographic***				
**Age**				
*65–74*	3188	200 (6.3)	1992	101 (5.1)
*75–79*	2470	221 (8.9)	2284	171 (7.5)
*80–84*	2281	312 (13.7)	2713	265 (9.8)
*≥ 85*	2683	650 (24.2)	4731	870 (18.4)
**Gender**				
*Female*	5725	812 (14.2)	9370	997 (10.6)
*Male*	4897	571 (11.7)	2350	410 (17.4)
**Marital Status**				
*Married*	5876	650 (11.1)	4791	502 (10.5)
*Single*	890	118 (13.3)	1239	158 (12.8)
*Divorced/separated/widow*	2976	521 (17.5)	4516	627 (13.9)
*Missing*	880	94 (10.7)	1174	120 (10.2)
***Clinical***				
**Major Comorbidities**				
*Cong. Heart Failure*	901	192 (21.3)	734	169 (23.0)
*Cardiac arrhythmias*	2001	378 (18.9)	645	128 (19.8)
*Valvular disease*	495	76 (15.4)	296	47 (15.9)
*Cardiac*	2754	491 (17.8)	1314	257 (19.6)
*Peripheral vascular disease*	639	98 (15.3)	335	72 (21.5)
*Hypertension*	4928	505 (10.2)	2088	240 (11.5)
*Neurological*	482	80 (16.6)	376	63 (16.8)
*Chronic pulmonary disease*	993	158 (15.9)	790	147 (18.6)
*Diabetes*	1965	241 (12.3)	892	130 (14.6)
*Renal Failure*	424	92 (21.7)	267	56 (21.0)
*Liver disease*	201	29 (14.4)	236	40 (16.9)
*Cancer*	672	175 (26.0)	556	116 (20.9)
*Anemia*	339	81 (23.9)	353	50 (14.2)
**Stroke Type**				
*Ischaemic*	9049	1148 (12.7)		
*Subarachnoid Haemorrhagic*	211	20 (9.5)		
*Haemorrhagic*	1362	215 (15.8)		
**Fracture Type**				
*Intracapsular*			5613	667 (11.9)
*Extracapsular*			5150	632 (12.3)
*Other/unspecified*			957	108 (11.3)
***Acute Care related***				
**Index LOS Category**				
< =*upper quartile*	8243	894 (10.8)	9152	1082 (11.8)
*> upper quartile*	2379	489 (20.6)	2568	325 (12.7)
**Admitted Stroke Unit at 1**^st^**S**				
*Yes*	483	43 (8.9)		
*No*	10139	1340 (12.5)		
**Surgery Procedure at 1**^st^**HF**				
*within 2 days*			2692	275 (10.2)
*after 2 days*			9028	1132 (9.7)
***Rehabilitation***				
**LHA Rehabilitation Rates**				
*-Overall,-Hospital (Q-I)*	2519	329 (13.1)	1640	186 (11.3)
*-Overall,+Hospital (Q-II)*	2196	308 (14.0)	3562	435 (12.2)
*+Overall,-Hospital (Q-III)*	2086	239 (11.5)	3311	383 (11.6)
*+Overall,+Hospital (Q-IV)*	3821	507 (13.3)	3207	403 (12.6)

Adjusted estimates of HRs with 95% CIs are summarized in Table 2. Several individual-level demographic, clinical and acute care-related factors are associated with increased risk of death during the 6-month follow-up period. Males (for hip fractures), patients previously hospitalised with diagnoses of severe disease, patients with haemorrhagic stroke or subarachnoid haemorrhage, and patients with longer LOS had an increased risk of death. Stroke patients admitted to stroke units and hip fractures undergoing surgery within 2 days from admission were associated to a decreased mortality.

**Table 2:** Results of Cox Multivariate Regression

***Dimension***	***Stroke***		***Hip Fracture***	
**Variable**	H.R. (95% c.i.)*	**P value**	H.R. (95% c.i.)*	**P value**

*Categories*				

***Demographic***				
**Age**				
*65–74 (R.C)*				
*75–79*	1.52 (1.31–1.78)	< 0.001	1.62 (1.30–2.03)	< 0.001
*80–84*	2.08 (1.79–2.41)	< 0.001	2.08 (1.68–2.57)	< 0.001
*≥ 85*	3.76 (3.27–4.32)	< 0.001	4.13 (3.39–5.01)	< 0.001
**Gender**				
*Females*	1.04 (0.94–1.14)	0.44	1.85 (1.65–2.07)	< 0.001
**Marital Status**				
*Married (R.C.)*				
*Single*	1.12 (0.95–1.31)	0.19	1.24 (1.04–1.46)	< 0.05
*Divorced/separated/widow*	1.12 (1.01–1.24)	< 0.05	1.25 (1.12–1.41)	< 0.001
*Missing*	0.96 (0.81–1.14)	0.64	0.98 (0.81–1.18)	0.80
***Clinical***				
**Major Comorbidities**				
*Cong. Heart Failure*	1.61 (1.36–1.90)	< 0.001	1.62 (1.25–2.10)	< 0.001
*Cardiac arrhythmias*	1.47 (1.22–1.78)	< 0.001	1.20 (0.95–1.53)	0.13
*Valvular disease*	1.07 (0.87–1.32)	0.53	1.21 (0.93–1.59)	0.16
*Cardiac*	0.78 (0.63–0.97)	< 0.05	1.03 (0.77–1.38)	0.83
*Peripheral vascular disease*	1.21 (1.02–1.43)	< 0.05	1.48 (1.18–1.85)	< 0.05
*Hypertension*	0.69 (0.63–0.76)	< 0.001	0.84 (0.73–0.95)	< 0.05
*Neurological*	1.05 (0.87–1.27)	0.58	1.38 (1.09–1.75)	< 0.05
*Chronic pulmonary disease*	1.15 (1.00–1.31)	< 0.05	1.18 (1.01–1.39)	< 0.05
*Diabetes*	1.11 (0.99–1.24)	0.07	1.22 (1.04–1.45)	< 0.05
*Renal Failure*	1.43 (1.20–1.70)	< 0.001	1.34 (1.06–1.70)	< 0.05
*Liver disease*	1.33 (0.97–1.80)	0.07	1.70 (1.28–2.24)	< 0.001
*Cancer*	2.17 (1.90–2.48)	< 0.001	1.80 (1.52–2.14)	< 0.001
*Anemia*	1.13 (0.93–1.37)	0.22	1.04 (0.82–1.33)	0.72
**Stroke Type**				
*Ischaemic (R.C.)*				
*Subarachnoid Haemorrhagic*	1.36 (1.03–1.79)	< 0.05		
*Haemorrhagic*	1.34 (1.19–1.50)	< 0.001		
**Fracture Type**				
*Intracapsular (R.C.)*				
*Extracapsular*			1.03 (0.93–1.14)	0.55
*Other/unspecified*			1.11 (0.93–1.32)	0.26
***Acute Care related***				
**Index LOS Category > third quartile**	2.93 (2.68–3.20)	< 0.001	1.27 (1.14–1.42)	< 0.001
**Admitted in Stroke Unit at at 1**^st^**S**	0.72 (0.55–0.94)	< 0.05		
**Surgery Procedure at at 1**^st^**HF within 2 days**			0.82 (0.72–0.93)	< 0.05
***Rehabilitation***				
**LHA Rehabilitation Rates**				
*-Overall,-Hospital (Q-I) (R.C.)*				
*-Overall,+Hospital (Q-II)*	1.07 (0.95–1.22)	0.27	1.13 (0.97–1.33)	0.12
*+Overall,-Hospital (Q-III)*	0.73 (0.64–0.83)	< 0.001	0.99 (0.84–1.16)	0.87
*+Overall,+Hospital (Q-IV)*	0.91 (0.82–1.02)	0.12	1.04 (0.89–1.22)	0.61

* longitudinal estimates from proportional hazards regression on the outcome of interest

Stroke patients resident in LHAs with a higher proportion of rehabilitated patients (quadrants III, IV) showed a reduced risk of death compared to subjects resident in LHAs with a lower proportion of rehabilitated patients, among whom also the use of in hospital rehabilitation was reduced (quadrant I). However, such reduction was small, not significant (HR 0.91, p-value 0.12) for patients resident in LHAs where most rehabilitation occurs in hospital (quadrant IV), while relatively high and statistically significant (HR 0.73, p-value < 0.01) for patients resident in LHAs where most rehabilitation occurs in extra-hospital settings (quadrant III). Among hip fractures, our data did not show any significant association between the average rehabilitation patterns found in LHAs and 6-month mortality.

## Discussion

According to our study, approximately 6,500 and 6,000 hospital admissions occur every year in Tuscany for first-ever strokes and hip fracture, matching results obtained using administrative databases [26,27] and population-based studies [28]. Similarly to other reports [29,30], we found that nearly 17% patients admitted for stroke and 3% of those admitted for hip fracture die during hospitalisation. Additional 15% approximately does not survive beyond 6 months following discharge.

The association found between demographic, clinical and acute care-related factors and 6-months mortality is consistent with recent studies and supports the validity of our data. A large European project in stroke [4] showed a more than two-fold risk of death for patients aged over 75, while another conducted in Australia reported age, pre-stroke disability and haemorrhagic stroke among the major determinants of stroke mortality at one year [31]. Reports from the UK [32] and US [9] on hip fractures similarly showed an increase in risk for age and males, and a Canadian study additionally also showed significance of pre-existent comorbidities [33]. The higher risk of diabetic patients among subjects with hip fractures confirms previous results [8]. The protective effect of hypertension could be due to over-reporting of this condition among cases presenting better conditions overall.

As far as acute care is concerned, our results are consistent with a meta-analysis showing a 17% one-year mortality reduction for stroke patients admitted in stroke units [34], a clear advantage very recently confirmed on field by a large observational follow-up study conducted on a sample of 11572 Italian acute stroke patients [35]. However, in Tuscany only 4% of the aged population affected by stroke is admitted to a stroke unit.

The mortality risk reduction found for hip fractures promptly operated after admission, inconsistently reported by others [36,37], is of interest, although it could be due to better overall conditions of patients eligible for prompt intervention.

Our study highlighted several important aspects related to rehabilitation services.

Overall, during the 6-months follow-up period, about 25% stroke and 45% hip fracture survivors used some kind of rehabilitative services. We found a high variation in the use of rehabilitation by LHA of residency, particularly for the fraction of rehabilitation provided in hospital.

The patterns of rehabilitation settings are quite consistent across the two acute events, reflecting the different availability of services provided by LHAs across the region, which in turn are the result of long and complex processes rather than of current evidence-based choices.

From an economic point of view, the high variability of expenditure found across LHAs raises great concern about equity issues around the provision of rehabilitation services. Similar differences in rehabilitation patterns appear in other European regions and can be very hard to modify.

Our results highlight the need for carefully assessing the cost-effectiveness of rehabilitation strategies for patients experiencing stroke and hip fracture. While reducing mortality is not the primary aim of rehabilitation services in general, massive practice of in-hospital rehabilitation was expected to show a protective effect at the population level. In our analysis, subjects with stroke – but not with hip fracture – are instead less likely to die during the six-month post acute period if they live in areas where actually a relatively large proportion of patients undergo rehabilitation services, but more often in extra-hospital settings.

The result cannot be fully explained with the available data, so that further investigations are needed to evaluate outcomes of expensive options such as in hospital rehabilitation, for which we still lack a clear evidence of survival improvement at the population level.

Finally, several limitations of our study are worth to be outlined.

Firstly, the validity of this report is strictly related to the secondary source used. Computerized health records may be still partially incomplete and some of the variation found may be due to the quality of data. To improve quality of this study, we excluded all records relative to one LHA not recording accurately.

Secondly, clinical information included in computerized records may not allow an adequate adjustment for the severity of the disease. This may be due partly to incorrect and/or incomplete clinical information, for which we have extended our data collection retrospectively to include all diagnoses available up to three years before the acute event. On the other hand, it can be virtually impossible to isolate the effect of individual characteristics that can be directly related to the health services provided. In our case, at the individual level, the relation between different rehabilitation treatment and 6-month mortality may be confounded by the tendency to offer specific treatment to patients characterized by specific clinical conditions that are in turn associated to the outcome. Consistently with the available literature [24], we have taken into account such "confounding by indication" by using an ecological treatment variable (LHA rehabilitation patterns), a valid solution if we make the reasonable assumption of a similar case-mix for stroke and hip fracture across LHAs.

Thirdly, statistical significance of the association found must be interpreted with caution for methodological reasons. The main results of the study are prone to the "ecological fallacy", i.e. some unmeasured individual characteristics, or other influential ecological confounders may be the actual determinants of the association found between rehabilitation patterns and mortality. To take into account this possible fallacy, we have used all information available related to the individual subject, at the same time providing an interpretation in terms of organizational factors that is highly plausible.

Fourthly, economic values are only indirectly estimated, based on the regional tariff lists, which are only a proxy of real health care costs. Furthermore, costs of outpatient services did not include pharmaceuticals. In the present study we have approached the economic analysis only through a rough estimate of the amount of resources spent for rehabilitation, that are certainly underestimated to some extent for the outpatient setting.

## Conclusion

There is a need for improved local policies for the optimal delivery of health services.

Our study, targeting two crucial conditions for elderly patients e.g. stroke and hip fracture, found a high variability of rehabilitation patterns across Tuscany, albeit coherent between the two pathologies, associated with remarkable differences in average expenditure. Differences in hazard rates for 6-month mortality after stroke at population level were also found, with significantly lower rates for those resident in areas where rehabilitation is provided often but in an extra-hospital setting.

The methodology presented in this report can be used and further refined to investigate population outcomes while comparing relative strengths and weaknesses of alternative rehabilitation strategies. However, these results need to be confirmed by a more robust information framework, in order to avoid possible ecological fallacy

A better assessment of rehabilitation policies requires the construction of composite indicators and the collection of *ad hoc *longitudinal data. More studies are needed to incorporate a range of outcomes properly measured, e.g. the improvement of functional status and the actual costs of health services over multiple diseases.

## Competing interests

The author(s) declare that they have no competing interests.

## Authors' contributions

FC designed the study and statistical analyses, initiating and coordinating the preparation of the manuscript; LR participated in the design of the study and preparation of the manuscript; PF participated in the design of the study and drafting the manuscript; RG participated in the design of the study and performed data transformation and all statistical analyses, developing all STATA software; FT participated in the design of the study and drafted the economic analysis; TDI performed systematic search of the literature; SB performed data extraction and computerized linkage from the regional database; EB conceived, designed and coordinated the study, and participated in drafting the manuscript.

All authors read and approved the final manuscript.

## Pre-publication history

The pre-publication history for this paper can be accessed here:


